# Editorial: Folded Synthetic Peptides for Biomedical Applications

**DOI:** 10.3389/fchem.2019.00448

**Published:** 2019-06-20

**Authors:** Jutta Eichler, Alessandro Contini

**Affiliations:** ^1^Department of Chemistry and Pharmacy, University of Erlangen–Nurnberg, Erlangen, Germany; ^2^Dipartimento di Scienze Farmaceutiche, Sezione di Chimica Generale e Organica “Alessandro Marchesini”, Università Degli Studi di Milano, Milan, Italy

**Keywords:** foldamer, bioactive peptide, conformational analysis, molecular switches, hydrogels

Folded peptides—and peptide motifs within proteins—are abundant in living organisms, where they are essential for the biological activities of the peptides and proteins. During the past decades, numerous research programs have been dedicated to understanding the rules that govern peptide folding. Simultaneously, a range of strategies have been established for the conformational stabilization of bioactive peptides, as well as for the *de novo* design of peptides with defined secondary structures. These methods are either based on the chemical modification of the peptide backbone, such as cyclization and side chain stapling, or on the use of a range of non-proteinogenic amino acids that, in a defined sequential arrangement, induce secondary structures in peptides. Such building blocks include D- and other non-proteinogenic amino acids, as well as β- and γ-amino acids.

This Research Topic comprises a collection of papers by an international group of 77 scientists with a background in synthetic, analytical, computational, and medicinal chemistry, as well as in biochemistry and pharmacology. Their research is presented here in a total of 11 papers (8 original research reports and 3 reviews), covering diverse aspects of folded synthetic peptides. These studies include the preparation and characterization of new peptide monomers with interesting folding properties, the synthesis and conformational analysis of non-natural peptides, as well as the use of folded peptidomimetics as molecular switches. Additionally, a range of biomedical applications, such as antimicrobial, anti-inflammatory, antiangiogenic, and immune-stimulating activities, are also reported.

Examples for the use of non-peptidic chemical moieties or non-natural amino acids for the generation of folded synthetic peptides include the use of 1,4-disubstituted 1*H*-1,2,3-triazoles (Schröder et al.) to mimic the amide bond. This bioisosteric replacement led to peptidodimetics whose foldamer properties were confirmed by both instrumental analysis and computational simulations. Similarly, two enantiomers of a bicyclic pyrrolidine-isoxazoline γ-amino acid were used to prepare diastereoisomeric model peptides (Oliva et al.). NMR, FT-IR, circular dichroism and molecular modeling studies confirmed that the (+) enantiomer was able to stabilize an α-turn conformation. Conversely,  Bucci et al. used syn-β2,3-diarylamino acids in conjunction with *S*-alanine for the generation of foldameric antiparallel β-sheets. As in the study by  Oliva et al., foldamer properties were confirmed here as well by combining instrumental analysis and theoretical calculations. Applications of synthetic peptides/peptidomimetics include their use as molecular switches, utilizing the light-triggered reversible shift from one conformation to another. In this context, Nuti et al. investigated the ability of a new photochromic azobenzene amino acid derivative to act as a conformational switch, when inserted into a model peptide.

A broad range of biopharmaceutical applications of synthetic peptides are also reported in this Research Topic. Recent applications of folded synthetic peptides targeting proteins of the outer membrane of gram-negative bacteria are reviewed by Robinson. In particular, synthetic β-hairpin mimetic peptides were found to interact with β-barrel and β-jellyroll domains in bacterial lipopolysaccharide transport and β-barrel folding machine complexes, thus representing a new frontier in the discovery of novel antimicrobial agents. Folded peptides are also reported to target tumor angiogenesis.  Zanella et al. present oligopeptides, designed by structural analysis and computational calculations, where Cα,α-disubstituted amino acids are exploited to stabilize the helical conformation that is essential to bind to the VEGF receptor at nanomolar concentration. Synthetic peptides as cancer-targeting immune system engagers (ISErs) are reported by Conibear et al. These molecule are generated using a range of chemoselective ligations, including copper-catalyzed azide-alkyne “click,” oxime, maleimide, and native chemical ligations. Furthermore, anti-inflammatory peptides targeting the interleukin-1 receptor (IL-1R) are reported by Geranurimi et al. The authors describe the structure-activity relationships of 12 peptides, in which different configurations of the α-amino-γ-lactam and β-hydroxy-α-amino-γ-lactam moieties were used to conformationally constrain an IL-1R peptide ligand.

This Research Topic also presents articles on the use of folded synthetic peptides for the design of functional biomaterials.  Hellmund and Koksch review the recent literature on the use of self-assembling peptides as mimics of the extracellular matrix. This is illustrated by peptide- and protein-based biomaterials that are able to support proliferation and differentiation of stem cells, demonstrating great potential of these peptides as tools in regenerative medicine. Applications of *N*-acetyl-β3-peptides to obtain innovative bio- and nanomaterials by supramolecular self-assembly are reviewed by Kulkarni et al. Compared to other organic and inorganic self-assembled systems, these foldamers show advantages in terms of biocompatibility, toxicity and functionalization potential. Last but not least,  Lammi et al. report a strategy to increase the stability and anti-diabetic properties of hempseed protein hydrolysates. This was achieved through encapsulation of hempseed hydrolysates into ionic self-complementary RADA16 peptide-based hydrogels. This study also evaluated the synergistic activity of RADA16-hemp hydrogels and sitagliptin, an orally available DPPIV inhibitor.

As a collection, the papers of this Research Topic demonstrate the broad impact of folded synthetic peptides on various fields of biological and biomedical research. It can be expected that research on folded synthetic peptides will continue to result in applications in medicinal chemistry and drug design, as well as the design of novel biomaterials.

## Author Contributions

AC and JE edited the articles of this research topic and wrote the editorial.

### Conflict of Interest Statement

The authors declare that the research was conducted in the absence of any commercial or financial relationships that could be construed as a potential conflict of interest.

